# Association between CTLA-4 gene polymorphisms and Type 1 diabetes in Kurdish patients

**DOI:** 10.3389/fendo.2026.1862007

**Published:** 2026-07-08

**Authors:** Hero H. Muhammed Saed, Gaza F. Salih, Hassan Mohammad Tawfeeq

**Affiliations:** 1 Chemistry Department, College of Education, University of Garmian, Kalar, Iraq; 2 Biology Department, College of Science, University of Sualimani, Slemani, Iraq; 3 Nursing Department, Kalar Technical Institute, Garmian Polytechnic University, Kalar, Iraq

**Keywords:** anti GAD antibody, CTLA-4, mRNA expression, SNP polymorphism, T1DM

## Abstract

Type 1 diabetes (T1D) is an increasingly complex disease influenced by both genetic and environmental triggers, leading to overactivity of the immune system. This preliminary study was conducted to investigate the association of two common cytotoxic T lymphocyte antigen-4 (CTLA-4) variants with T1D susceptibility in the Kurdish population of Iraq. Additionally, we evaluated the correlation between these polymorphisms and anti-GAD antibody positivity, as well as their possible influence on CTLA-4 gene expression. In this study, 52 patients (28 males and 24 females) with T1D and 21 healthy control subjects were genotyped for two (CTLA-4) SNPs, A>G (rs231775) and -318 C>T (rs5742909), using direct DNA sequencing. Furthermore, serum anti-GAD antibody levels were measured by ELISA technique, and the expression of CTLA-4 levels was assessed with quantitative real-time PCR. We discovered that the A>G (rs231775) variant was significantly associated with T1DM. The G allele frequency was significantly higher in type 1 diabetes patients (36.5%, P = 0.0188). The -318 C/T polymorphism showed no significant differences between groups. The GG genotype of the +49A/G polymorphism exhibited a greater prevalence in anti-GAD positive patients relative to negative individuals (77.78% vs. 22.22%), indicating an elevated risk trend (OR = 2.77, 95% CI: 0.51–14.91); however, this finding did not achieve statistical significance (P = 0.28). The (CTLA-4) mRNA gene expression was observed to be non-significantly elevated in T1D relative to the control group (p = 0.1239). Our data indicate that the G allele of the CTLA-4 + 49 A>G variant is associated with increased T1D susceptibility in the Kurdish population, while the genotypic association showed a nominal trend.

## 1 Introduction

Type 1 diabetes (T1D) is a persistent autoimmune disease arising from the interplay of hereditary and environmental influences. These elements collectively initiate a vigorous autoimmune attack on pancreatic β-cells, resulting in their progressive destruction (1). The disease manifests due to the inheritance of a genetically predisposed adaptive immune system that reacts to β-cell antigens. In this case, autoreactive T cells that have evaded central tolerance induce the death of β-cells via cytokine-mediated pathways (2). A key immune checkpoint molecule involved in regulating T cell activity is (CTLA-4) cytotoxic T lymphocyte antigen-4. Identified in 1996 as a primary susceptibility gene for type 1 diabetes, it is situated on chromosome 2 (2q33) and comprises 4 exons and 3 introns (3). This immunoglobulin superfamily member expressed on T lymphocytes and serves as a negative regulator of T cell activation, which exhibits a comparable binding affinity for the costimulatory receptors B7-1 (CD80) and B7-2 (CD86) on antigen-presenting cells (APC), and this association generates an inhibitory signal that suppresses T cell activation (4). The CTLA-4 gene harbors several single nucleotide polymorphisms (SNPs) that may impact its expression, resulting in amino acid alterations and modifications in mRNA splicing, potentially affecting T cell function and diminishing the immunological response. A prevalent variation in the coding region of the CTLA4 gene is A>G (rs231775), which has been documented for its functional impact on gene expression and protein efficacy, leading to the substitution of alanine (Ala) with threonine (Thr) in the first exon of the signal peptide (5). The +49A/G polymorphisms affect the glycosylation of CTLA-4 and its intracellular/surface distribution. This leads to diminished levels of CTLA-4 (Ala17) on the cell surface, potentially elucidating the decreased inhibitory effect of CTLA-4 observed in +49G allele carriers (6).

Another well-researched polymorphism marker that has garnered a lot of interest is a C-to-T substitution at the promoter region’s position -318 C>T (rs5742909). Which causes the elevation of transcriptional activity. It has been discovered that the T allele is linked to higher promoter activity than the C allele and to noticeably higher production of both cell-surface CTLA-4 on activated cells and CTLA-4 mRNA in unstimulated cells (7).

These two SNPs have been investigated in several previous studies with type 1 diabetes, but with inconclusive findings. As mentioned above the -318 C>T (rs5742909) promoter variant has been confirmed to be associated with an increased transcriptional activity. Thus, this preliminary study aimed to investigate the potential association of these two common polymorphisms (CTLA-4 gene SNPs, A>G (rs231775) and -318 C>T (rs5742909) with T1D susceptibility in the Kurdistan population. Additionally, we assessed the relationship of these variants with anti-GAD antibody positivity and their potential impact on CTLA-4 gene expression.

## 2 Materials and methods

### 2.1 Study population and design

The current preliminary study enrolled a total of 52 patients diagnosed with type 1 diabetes mellitus (T1DM), comprising 28 males and 24 females (mean age 15.34 ± 4.80), who were recruited from Kalar General Hospital, Kurdistan, Iraq, along with 21 age-matched nondiabetic control participants (14.88 ± 4.26). The diagnosis of T1D was established in accordance with the recommendations of the International Society for Pediatric and Adolescent Diabetes (ISPAD). All patients had a disease duration of more than one year and were receiving insulin therapy (a twice-daily premixed insulin regimen), and all blood samples were systematically collected during morning fasting before the patients received their scheduled insulin dosage to ensure baseline expression analysis. Demographic data, clinical parameters (FBS, C-peptide, insulin, HbA1c), associated complications, and the existence of other autoimmune disorders were documented using a structured questionnaire. The participants, including patients and controls, were of Kurdish ethnicity. The study protocol received approval from the Ethics Committee of the Research Center at the University of Garmian, Kurdistan Region (Approval No. 1115; October 24, 2024). And the research was executed according to the principles of the Declaration of Helsinki.

### 2.2 Serological test

Fasting blood glucose and glycosylated hemoglobin (HbA1c) levels were assessed using enzymatic methods with the variant hemoglobin A1c program on the Cobas C111 analyzer. The serum C-peptide level was determined by Cobas E411 system. In addition, anti–glutamic acid decarboxylase (anti-GAD) antibodies were detected using a quantitative enzyme-linked immunosorbent assay (ELISA) technique, supplied by Medipan GmbH, (Wendelsheim, Germany). All assays were performed in accordance with the manufacturers’ instructions.

### 2.3 DNA extraction

About three mL of blood specimen was obtained from each participant in ethylenediaminetetraacetic acid (EDTA) tubes for the extraction of nucleic acid. DNA was extracted using the TransGen Biotech Genomic DNA Extraction Kit (China) according to the manufacturer’s protocols. The quantity and purity of the isolated DNA were measured spectrophotometrically using a NanoDrop instrument (Thermo Scientific, Wilmington, DE, USA).

### 2.4 DNA sequencing

Two polymorphic loci of the CTLA-4 gene were studied: a variant in exon 1 at +49A/G (rs231775) and promoter variant at a -318C/T (rs5742909). A specific single primer pair that flanks both polymorphic sites was used to amplify both loci, resulting in an 815 bp amplicon. F 5′-GCAGCTTCTTTTCCGCCTA-3′ and R 5′-CTCCTCCATCTTCATGCTCC-3′. Add Bio Meditek Hot Start Taq DNA Polymerase Master Mix kit was used for PCR amplification, comprising Taq DNA polymerase, dNTPs, MgCl_2_, and reaction buffers. The PCR reaction system included 10 μl of 2X PCR Master Mix (final concentration of 1X), 1 μl of each primer (10 pmol/μl), 3 μl of genomic DNA 35 ng, and 5 μl of nuclease-free water to a total reaction volume of 20 μl. PCR was carried out in a PCR thermocycler (Eppendorf, Mastercycler gradient, Germany). Using the following procedures: initial denaturation at 95 °C for 10 mins, followed by 35 amplification cycles. Each cycle consisted of denaturation at 95 °C for 20 sec, annealing at 56 °C for 30 sec, and extension at 72 °C for 20 sec. The PCR product was analyzed using 1% agarose gel electrophoresis. Subsequently, 16 μL of each PCR product, together with the forward primer, was submitted to Macrogen (South Korea) for direct sequencing. Sequencing was conducted using the Sanger technique on an ABI 3730 DNA sequencer (Macrogen, South Korea). The produced nucleotide sequences were analyzed with the Chromas version 2.4 software and BLAST tools.

### 2.5 RNA extraction

Total RNA was isolated from whole blood samples obtained from all participants (patients and control group) using GeneAll Biotechnology’s GeneAll Hybrid-R Blood RNA kit (China), in accordance with the manufacturer’s information. The purity and quality of extracted RNA were evaluated by measuring the absorbance ratio at 260/280 nm, with acceptable values ranging from 1.8 to 2.0. All RNA samples were stored at -86 °C until further analysis.

### 2.6 Quantitative real-time PCR analysis

Relative CTLA-4 gene expression analysis was carried out using a C1000 Touch™ Thermal Cycler (Bio-Rad Laboratories, Hercules, CA, USA). One-step RT-qPCR was carried out using AddGreen RT-qPCR Master (Addbio, ADDBIOMEDITEK Co., Ltd., South Korea; Cat. No. 71302) in accordance with the manufacturer’s guidelines. In 96-well plates using the following comprising conditions. Reverse transcription at 50 °C for 20 min, initial denaturation at 95 °C for 10 min, followed by 40 cycles of denaturation at 95 °C for 15 sec and annealing at 58 °C for 1 min. Melting curve analysis was performed from 65 °C to 95 °C to confirm amplification specificity and recorded using 3 technical replications. Primers used for CTLA-4 amplification were 5′-TATCCAAGGACTGAGGGCCA-3′ (forward) and 5′-ACATTCTGGCTCTGTTGGGG-3′ (reverse). The ACTB (β-actin) gene was used as the endogenous reference control. Each 20 µL reaction mixture comprised 10 µL of SYBR Green Master Mix, 0.8 µL of each primer 10 pmol/µL, 2.4 µL of total RNA, and 6 µL of nuclease-free water. A negative control (NTC) was used in each run. Equal amounts of RNA were utilized in all reactions. The 2^-^ΔΔCT technique was employed for relative CTLA-4 gene expression analysis (8).

### 2.7 Statistical analysis

The distribution of genotypes for both polymorphisms (+49 A/G and -318 C>T) was assessed for deviation from the Hardy-Weinberg Equilibrium (HWE) in the control group using the Chi-square testing. Fisher’s exact test based on a 2 × 2 was used for analyzing the genotype and allele frequencies contingency tables (presence vs. absence). This method was applied to compare patients with healthy controls, as well as anti–GAD–positive (greater than 5 U/mL) and anti–GAD–negative (less than 5 U/mL) patients with type 1 diabetes. The strength of associations was found by calculating odds ratios (ORs) with corresponding 95% confidence intervals (CIs). In order to correct for multiple comparisons, P values < 0.05/n were considered significant, where n represents the number of comparisons (Bonferroni). Statistical comparisons between healthy controls (n = 9) and patients (n = 25) were conducted using ΔCt values. Data normality was measured using the Shapiro–Wilk test. As ΔCt values did not consistently satisfy the assumptions of normal distribution, group comparisons were conducted utilizing the two-tailed Mann–Whitney U test. Relative CTLA-4 gene expression levels were determined using the ΔΔCt technique and represented as fold changes (2^-^ΔΔCt) for graphical presentation only. All statistical analyses were conducted using GraphPad Prism software (version 10.0.2; GraphPad Software, San Diego, CA, USA). A p-value < 0.05 was considered statistically significant.

## 3 Result

The study population, demographic, and biochemical characteristics (patients vs control group) are presented in **Table 1**. All patient cohort utilized a twice-daily premixed insulin regimen, and. Both groups were matched based on age and BMI. Individuals with T1DM demonstrate markedly elevated FBS and HbA1c levels in comparison to the control group, with mean values of 325 mg/dL and 9.81%, respectively (both P < 0.0001). Conversely, serum C-peptide levels were significantly diminished in the diabetic group compared to controls group (P < 0.0001). In addition, anti–glutamic acid decarboxylase (anti-GAD) antibodies were detected in 59.61% of patients with T1DM, with a mean concentration of 18.84 ± 21.15 U/mL, which was substantially greater than that observed in the control (2.51 ± 0.99 U/mL; P = 0.0003).

**Table 1:** Demographic and biochemical features of patients with T1D and the control group.

Biochemical characteristic	Type1D (n = 52)	Control (n = 21)	P-value
Gender			
Female	24 (46%)	10 (48%)	
Male	28 (54%)	11 (52%)	
Mean age (Years)	15.34 ± 4.80	14.88 ± 4.26	0.684
BMI (Kg/m2)	21.32 ± 6.60	21.49 ± 4.67	0.915
Family history of type 1 diabetes	None	None	–
Diabetes duration (years)	6.13 ± 5.16	-	–
Insulin dependent	All (Twice-daily premixed)	None	–
Fasting blood glucose (mg/dL)	325.0 ± 143.4	92.71 ± 14.93	<0.0001****
HbA1c (%)	9.81 ± 2.66	4.68 ± 0.42	<0.0001****
Fasting C-peptide (ng/mL), Median [IQR]	0.0090 [0.003-0.07]	3.715[1.93-5.44]	<0.0001****
Anti-Gad (U/mL)	18.84 ± 21.15 (59.61%)	2.51 ± 0.99	0.0003***

BMI, body mass index; HbA1c, Glycated hemoglobin; Anti-Gad, Glutamic Acid Decarboxylase Autoantibodies; IQR, Interquartile Range.

***, Highly significant; **** , Extremely highly significant.

### 3.1 Association of CTLA-4 SNPs with susceptibility to T1D

This study was conducted to investigate the association between two common SNPs in the CTLA-4 gene, +49 A>G (rs231775) and -318 C>T (rs5742909), and their potential role in type 1 diabetes (T1D). PCR amplification of the CTLA-4 gene fragment was confirmed by agarose gel electrophoresis. The genotypic and allelic distributions of both variants are summarized in **Table 2**. The genotype distributions of both variants in the control group were in strict compliance with the Hardy–Weinberg Equilibrium (HWE), with p-values of 0.511 and 0.911, respectively.

**Table 2:** Genotype and allele frequency of (+49 A>G and −318 C>T) SNPs in T1DM.

Genes	Genotypes alleles	Patients (%) n = 52	Controls (%) n = 21	HWE (p-value) of control	Odd ratio (95% CI)	95% CI	P-value
rs231775 A>G (+49AG)	AA	23 (44)	15 (71)	0.511	1 (Ref)		_
	AG	20 (38)	5 (24)		2.61	0.81–8.42	0.108 (NS)
	GG	9 (17)	1 (5)		5.87	0.67–51.1	0.109 (NS)
Allele	A	66 (63.5%)	35 (83.3%)		0.35	0.14–0.86	0.0188*
	G	38 (36.5%)	7 (16.7%)		2.88	1.17–7.11	0.0188*
rs5742909 C>T (-318 CT)	CC	48(92)	20(95)	0.911	1 (Ref)		_
	CT	4 (8)	1 (5)		1.67	0.17 –16.0	0.65
	TT	0	0		–	–	–
Allele	C	100 (96.2%)	41 (97.6%)		0.61	0.07–5.62	1
	T	4 (3.8%)	1 (2.4%)		1.64	0.18–15.12	1

N, number of participants; %, percentage; CI, 95% Confidence Interval; P, Probability; p < 0.025 is considered statistically significant; chi-squared test (χ ^2^) for genotype and *allele frequencies.

To account for multiple testing, a Bonferroni correction for two SNPs was applied, setting the significance threshold at P < 0.025.

**Figure 1:**
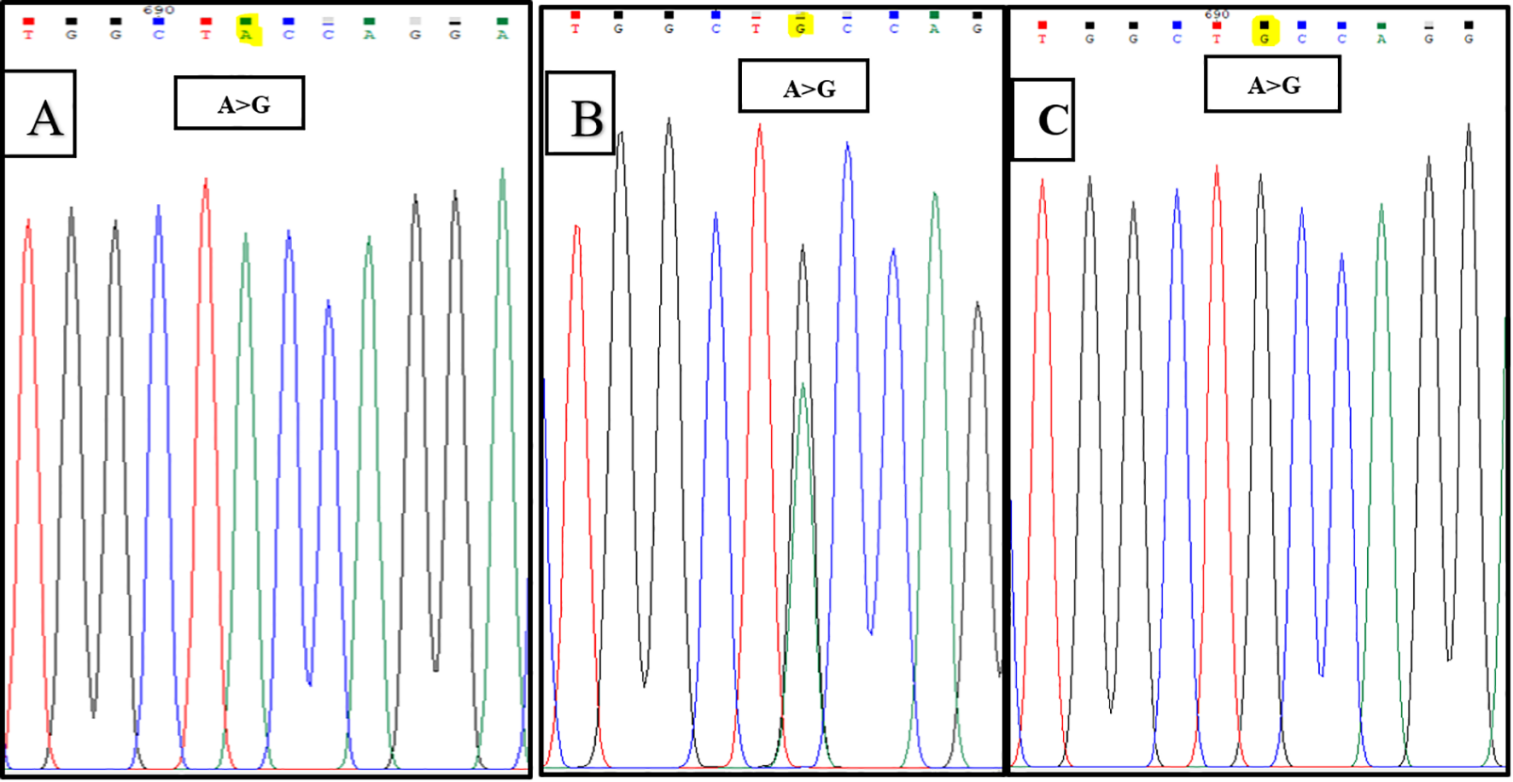
Representative sanger sequencing chromatograms of CTLA-4 gene polymorphisms. Rs231775 (+49 A>G) showing **(A)** AA, **(B)** AG, and **(C)** GG genotypes.

For the +49 A>G (rs231775) variant, the wild type AA genotype was more frequent among healthy controls than among the patient group (71% vs. 44%) (**Figure 1**). Compared to the AA group, the AG and GG genotypes showed an increased risk trend for T1D with ORs of 2.61 (95% CI: 0.81–8.42) and 5.87 (95% CI: 0.67–51.1), respectively, although these did not reach statistical significance after Bonferroni correction (P > 0.025).

In contrast, allelic frequency analysis demonstrated a statistically significant difference between the two groups. The G allele was significantly more frequent in T1D patients than in healthy controls (36.5% vs. 16.7%), indicating increased susceptibility to T1D among carriers of this allele (OR = 2.88, 95% CI: 1.17–7.11, P = 0.0188). Conversely, the A allele demonstrated a significant protective effect (OR = 0.35, 95% CI: 0.14–0.86, P = 0.0188).

**Figure 2:**
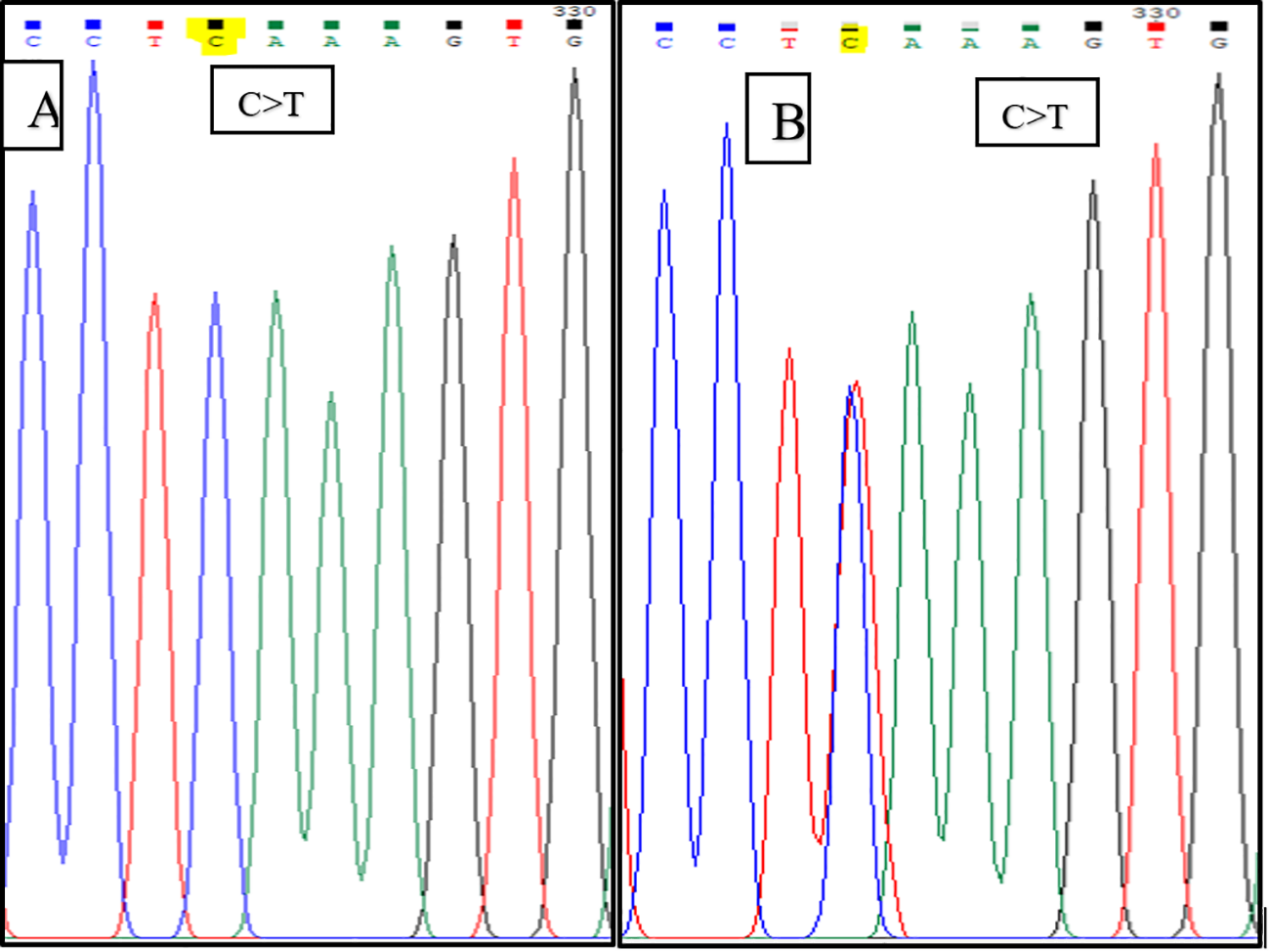
Representative sanger sequencing chromatograms of CTLA-4 gene polymorphisms. Rs5742909 C>T (-318 CT) showing **(A)** CC, and **(B)** CT, genotypes.

Analysis of the −318 C>T polymorphism observed no statistically significant differences in either genotypic or allelic frequencies between individuals with T1D and healthy controls (p < 0.025), indicating that this variant is not associated with susceptibility to T1D in the studied population (**Figure 2**).

### 3.2 Association of CTLA-4 SNPs with anti-GAD Ab status

The association between CTLA-4 gene SNPs (+49 A>G and −318 C>T) and anti–glutamic acid decarboxylase was further evaluated. Patients were stratified according to anti-GAD antibody positivity (>5 U/mL). Data analysis of the CTLA-4 + 49 A>G polymorphism revealed no statistically significant association with anti-GAD antibody positivity among Kurdish patients with type 1 diabetes (p < 0.05). The prevalence of positive GAD-Ab tended to be higher for the GG genotype (77.8% positive vs. 22.2% negative); however, the difference not achieve statistical significance (p = 0.28). Allelic analysis showed a similar pattern, with the G allele more prevalent in the anti-GAD–positive group than in the negative group (68.4% vs. 31.6%), but this difference was also not statistically significant (p = 0.21).

Similarly, evaluation of the CTLA-4 −318 C>T polymorphism demonstrated no statistically significant association with anti-GAD antibody status. Neither the CC nor the CT genotype showed a significant relationship with antibody positivity (CC: OR = 0.47, 95% CI: 0.05–4.82, p = 0.64; CT: OR = 2.14, 95% CI: 0.21–22.13, p = 0.64). In agreement with the genotypic results, allelic analysis revealed no significant association for either the C allele (OR = 0.48, 95% CI: 0.05–4.77, p = 0.65) or the T allele (OR = 2.08, 95% CI: 0.21–20.75, p = 0.65) (**Table 3**).

**Table 3:** Association of +49 A>G and −318 C>T SNPs with anti–GAD status in patients with T1D.

CTLA-4 + 49A/G Genotype	Total per Genotype	Positive (>5 U/ml) n=31	Negative (< 5 U/ml) n=21	Odds Ratio (OR)	95% CI	P-value
AA	23	12 (52.17%)	11 (47.83)	0.57	0.19 – 1.76	0.14
AG	20	12 (60%)	8 (40%)	1.03	0.33 – 3.21	1
GG	9	7 (77.78%)	2 (22.22%)	2.77	0.51–14.91	0.28
A		36 (54.55%)	30 (45.45%)	0.55	0.23 – 1.30	0.17
G		26 (68.42%)	12 (31.58%)	1.81	0.78 – 4.17	0.21
-318 C>T						
CC	48	28 (58.33%)	20 (41.67%)	0.47	0.05 – 4.82	0.64
CT	4	3 (75%)	1 (25%)	2.14	0.21 – 22.13	0.64
TT	0	0	0	—	—	—
C		59	41	0.48	0.05 – 4.77	0.65
T		3	1	2.08	0.21–20.75	0.65

The percentages represent the proportion of positive/negative cases within each specific genotype group (row-wise calculation).

Additionally, the study did not reveal statistically significant between both genetic variants and any of the analyzed clinical biomarkers, including FBS, HbA1c, and C-peptide.

### 3.3 CTLA-4 expression analysis

**Figure 3:**
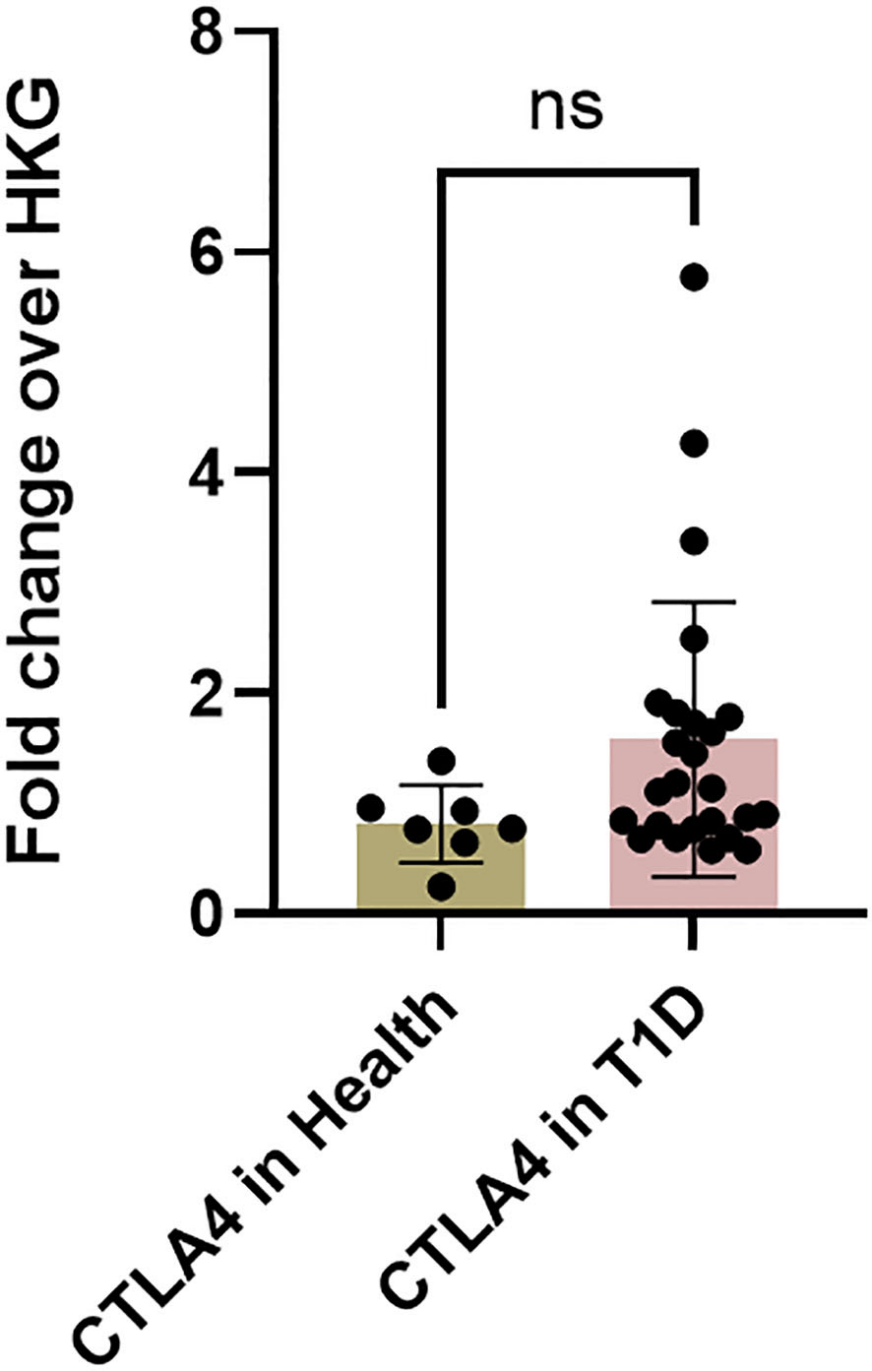
Relative expression of the CTLA-4 gene normalized to the housekeeping gene ACTB (mean ± SE) in T1D patients and control group (p = 0.1239).

Relative quantification of CTLA-4 gene expression was assessed using quantitative real-time PCR (q-RT-PCR) in 25 patients with T1D and 9 normal healthy control individuals. The CTLA-4 mRNA level was slightly higher in the T1D group (mean = 1.58 ± 1.24) compared to controls (mean = 0.8199 ± 0.3475), despite the absence of statistical significance in this difference (**Figure 3**).

## 4 Discussion

In this preliminary case-control study, we examined the potential association between two CTLA-4 single nucleotide polymorphisms (SNPs), rs231775 (+49 A>G) and rs5742909 (−318 C>T), and the susceptibility to T1D in the Kurdish population of Iraq. For the +49 A>G polymorphism, a strong positive association was observed for the G allele (p < 0.025). These findings are consistent with various case reports or systematic reviews connected to increased risks of type 1 diabetes in several populations including, Iranian/Kurdish (9), Egyptian (10), Sudan (11), Ethiopian (12), Estonian (13), Belgian (14), Germany (15), Spanish, French, Italian, and Mexican-American (16). Typically, our findings are supported by a large meta-analysis conducted by Chen et al. (2013), which analyzed 14 cohort studies involving 3,959 participants; the CTLA-4 + 49 G allele was recognized as a substantial risk factor for T1DM (OR = 1.11, 95% CI: 1.01–1.22, p = 0.03) (17). Similarly, Wang et al. (2014) (18), in a comprehensive investigation of 58 international studies including 30,723 T1DM cases and 45,254 controls, reported a modest overall correlation between the CTLA-4 + 49 G allele and T1DM across European (Caucasian), Asian, and Middle Eastern populations. Although the magnitude of this association varied—being strongest in European and Asian populations and more heterogeneous in Middle Eastern and Latino groups—the global evidence strongly supports a role for the G allele in increasing autoimmune susceptibility (OR = 1.42, p < 10^-^^5^). In contrast, no significant association between the CTLA-4 + 49 polymorphisms and T1DM has been reported in several populations, including Iraqi (19),Turkish (20), Azerbaijani (21), Jordanian (22), Egyptian (23), Portuguese (24), Brazilian (25), Korean (26), Southern Brazilian (27), and Chilean (28). The discrepancy results obtained from previous studies may be due to differences in ethnic and genetic background, geographical region, environmental factors, various experimental designs and samples sizes that could impact results. A recent meta-analysis by Tuerxunyiming et al., aggregating data from 19 case-control studies involving 6,358 participants, demonstrated a significant association between the +49A/G SNPs and type 1 diabetes risk. However, a significant association was observed in Asian ethnicity after subgroup analysis, but this was not observed in Europeans (29). For −318 promoter polymorphism, our findings indicate that the allele and genotype frequency distributions did not differ significantly between patients with T1DM and healthy controls. Several studies have confirmed our results among different populations, including Iraqi (30), Azerbaijanian (31), Tunisian (32), Jordanian (22), Korean (26), Northern China (33),Chilean (34), South Brazile (27) diabetic patients. Additionally, our results are further supported by the comprehensive HuGE review and meta-analysis conducted by Kavvoura and Ioannidis (2005), which analyzed 33 studies from populations worldwide and reported the −318 C>T (rs5742909) promoter variant presents a much more complex and often non-significant risk for type 1 diabetes (35). Unlike our result, the finding from a meta-analysis by Chen and Li (2019), who analyzed 76 studies, reported that the −318 polymorphism was associated with T1D risk factors in Caucasian and South Asian populations (36). Consequently, our findings validated that -318 T/T homozygotes are rare within populations, and the -318 T mutation may be regarded as protective against increased T cell activation and autoimmune diseases (22). These inconsistent findings may be attributed to genetic heterogeneity among populations, differences in allele frequencies, or the absence of specific variants in certain ethnic or geographical groups.

Additionally, our investigation between the +49A/G and -318 polymorphisms with anti-GAD antibody positivity, demonstrates that neither CTLA-4 + 49 A/G nor −318 C>T polymorphisms were significantly associated with GAD-Ab positivity. Regarding CTLA-4 + 49 A/G, the frequency of the GG genotype was higher in the diabetic group with positive GAD-Ab (> 5 U/ml) (77.78%) than in the GAD-Ab negative subjects (22.22%), but this increase did not reach statistical significance (P = 0.28). Despite the lack of significance, individuals carrying the GG variant exhibited a higher odds ratio (OR = 2.77). These results align with the study conducted by Chen et al. (2017), which examined antibody profiles and found no significant association with GAD-Ab, despite finding a link with IA-2A. This suggests that the CTLA-4 + 49G/A SNP affects the autoimmune profile differently across various ethnic groups (37).

In contrast, our findings differ from Hayashi et al. ‘s result, who reported a significant link between the CTLA-4 + 49 GG genotype and (67% vs. 39%) positive anti-GAD antibody status in Japanese and proposed that CTLA-4 polymorphisms might affect autoantibody expression and immunological heterogeneity, rather than directly determining T1DM risk (38). Similar results were achieved by Mochizuki et al. (2003),who highlighted that the G allele of CTLA-4 + 49A/G was more common in Japanese children with high anti-GAD antibody levels, suggesting that this polymorphism may influence autoimmune intensity (32).

The inhibitory function of CTLA-4 in immune regulation underscores the importance of factors that modulate its expression and activity. While single nucleotide polymorphisms (SNPs) may not directly cause disease, they can act as significant risk modifiers by altering gene expression and the function of the encoded protein.

Despite the functional impact of the genotypes, our analysis indicates that the +49A/G polymorphism did not exhibit substantial influence of gene expression levels in the case-control studied (P = 0.1239). While a trend towards heightened expression was noted in patients compared to controls, this increase was not unequivocally associated with any particular genotype. This suggests that in the Kurdistan community, the +49A/G SNP may not be the primary factor influencing CTLA-4 mRNA variability; instead, other regulatory elements and environmental variables may exert a more substantial effect on the gene’s transcriptional activity. The up-regulation of CTLA-4 in the patients’ individuals is consistent with previous reports linking elevated CTLA-4 expression to clinical outcomes in various autoimmune diseases, including Hashimoto’s Thyroiditis (39), myasthenia gravis (MG) and multiple sclerosis (MS) (40), and systemic lupus erythematosus (SLE) (41). While different studies have shown that the Thr to Ala substitution of the rs231775 G allele causes lower mRNA efficiency and lower CTLA-4 when compared to the rs231775 A allele (6). Our results align with the compensatory model proposed by Ligers et al. (2001) (40). and the observations of Wang et al. (2011) (42), who noted increased CTLA-4 expression in active autoimmune environments. In which the immune system, sensing the functional instability and poor surface expression of the 17-Alanine (G-allele) protein, paradoxically increases mRNA production in a failed attempt to restore immune regulation. The mechanisms of CTLA-4 expression and regulation are complex, requiring additional research to clarify how CTLA-4 expression affects T cell activity.

Despite the significant findings, several limitations must be acknowledged. First, the preliminary nature of this study is reflected in the small sample size and its single-center design, which may limit the generalizability of the results. Second, the gene expression analysis was conducted on a smaller subset of the cohort due to stringent sample quality requirements. Additionally, qRT-PCR was performed using whole blood instead of isolated PBMCs or T-cells, which may affect the specificity of the expression data. These limitations highlight the need for future large-scale, multi-center investigations to validate these findings and provide more comprehensive genetic data on the Kurdish population.

## 5 Conclusion

This is the first preliminary study to investigate the role of CTLA-4 polymorphisms in the risk of developing T1D within the Kurdish population of Iraq. While prior research (Al-Isawi et al., 2022) evaluated this variant in broader Iraqi pediatric cohorts, our study highlights a distinct ethnic group characterized by unique geographic, lifestyle, and regional environmental factors. Although definitive genomic distinctions between Iraqi and Iranian Kurdish populations require further genome-wide verification, our findings underscore the importance of region-specific screening to better understand the genetic architecture of T1DM within localized Middle Eastern communities. Our results indicate that while the +49 A/G (rs231775) allelic frequency is significantly associated with T1D development, the genotypic association showed only a nominal trend. Furthermore, the −318 C>T variant did not show a significant association. Although the +49 GG genotype exhibited a trend toward higher GAD-Ab levels and increased CTLA-4 mRNA expression, these results did not reach statistical significance. These findings suggest that while specific CTLA-4 alleles influence T1D susceptibility, the underlying regulatory mechanisms remain to be elucidated in larger, multi-center cohorts. The findings of the present study should be interpreted cautiously due to the relatively small sample size. Therefore, further large-scale studies with larger and more balanced cohorts are required to validate these preliminary findings and to establish more robust conclusions regarding the association between these SNPs and disease susceptibility.

## Data Availability

The original contributions data presented in the study are not publicly available due to patient confidentiality and ethical restrictions. Further inquiries can be directed to the corresponding author.
